# The Cerebellum in Poststroke Movement Disorders: A Case of Head Tremor Indicating a Cerebrovascular Emergency

**DOI:** 10.1155/crnm/9145358

**Published:** 2025-09-02

**Authors:** Aimen Vanood, Ruth H. McConnell, Bart M. Demaerschalk

**Affiliations:** ^1^Department of Neurology, Mayo Clinic College of Medicine and Science, Phoenix, Arizona, USA; ^2^Department of Radiology, Mayo Clinic College of Medicine and Science, Phoenix, Arizona, USA

**Keywords:** case report, movement disorder, stroke, tremor

## Abstract

Tremor is a common symptom encountered in outpatient practice, particularly within a neurology movement disorder clinic. However, tremor can also be pertinent to inpatient medicine and can warrant emergent evaluation. We describe a case of a 72-year-old female who developed acute onset postural head tremor without appendicular tremor during a hospital admission for chest pain and leukostasis. This case explores the localization of tremor and the differential diagnosis of head tremor specifically. Additionally, this report serves as a reminder to consider ischemic stroke in the diagnostic evaluation of acute onset neurological symptoms.

## 1. Introduction

Acute, or apoplectic, onset of neurological symptoms typically implicates a vascular etiology, such as acute ischemic stroke. Traditional stroke symptoms include unilateral hemiparesis, hemianopsia, and aphasia [1]. However, atypical stroke presentations can also occur, including hyperkinetic or Parkinsonian movement disorders. Hemiballismus and hemichorea are the most common poststroke movement disorders and can occur due to lesions of the subthalamic nucleus, or, less commonly, involving the caudate, putamen, or thalamus [2]. Poststroke tremor is generally uncommon; however, strokes involving the brainstem and cerebellum can result in palatal tremor or appendicular Holmes tremor [3].

Current guidelines recommend emergent evaluation for patients presenting with symptoms concerning for acute ischemic stroke, to determine candidacy for thrombolysis and thrombectomy [4, 5]. While protocols exist and have been streamlined for patients presenting to the emergency department with stroke-like symptoms, in-hospital stroke can be associated with delays in identification and subsequent intervention [6]. Atypical stroke presentations can further contribute to this delay. We present a case of an in-hospital stroke presenting with acute onset head tremor, with the goal of raising awareness of this uncommon stroke presentation, and emphasizing the importance of prompt evaluation in patients developing acute neurological symptoms.

## 2. Case Report

A 72-year-old right-handed female with a history of remote atrial fibrillation (not on anticoagulation), unspecified leukemia, coronary artery disease with stenting, and recent non-ST elevation myocardial infarction (NSTEMI) with consequent ischemic cardiomyopathy, hypertension, hyperlipidemia, Type 2 diabetes mellitus, and remote breast cancer presented to the hospital due to chest pain four days after percutaneous coronary intervention with stenting. She was on dual antiplatelet therapy with aspirin and clopidogrel at the time of admission. Laboratory values revealed significant leukocytosis (white blood cell count over 220 × 10^9^/L); she was ultimately diagnosed with chronic myelogenous leukemia (CML) with leukostasis. Diagnostic testing did not reveal any evidence of in-stent restenosis or stent thrombosis, and her chest pain improved spontaneously. During admission, she developed acute onset head tremor, prompting stroke neurology engagement. She also endorsed mild vertiginous sensation but no other neurological symptoms. She had no personal or family history of tremor. She denied any muscle spasms. She was not taking any medications associated with tremor. The patient's National Institutes of Health Stroke Scale (NIHSS) was 0. However, on neurological exam, the patient exhibited a postural, high amplitude, low frequency, yes-yes head tremor without evidence of entrainment nor resolution with distraction. The tremor resolved with sleeping and was provoked by sitting up and standing. There was no appendicular rest, postural, or kinetic tremor, myoclonus, or hemiballismus, and the patient exhibited normal tone and no bradykinesia, as well as intact strength, deep tendon reflexes, and sensation. There was no evidence of cervical dystonia. There was no appendicular or truncal ataxia. Cranial nerve exam revealed intact visual fields, normal extraocular movements without nystagmus or vertical gaze palsy, negative test of skew, no impairment in facial sensation or facial strength, intact hearing, no palatal tremor, and no tongue deviation. There were no impairments in consciousness, cognition, or language.

Noncontrast computed tomography (CT) of the head revealed no evidence of infarction, intracerebral hemorrhage, or structural lesions within the brain. The patient was not a candidate for thrombolysis given the interval between the recognition and reporting of clinical symptoms exceeded the 4 ½ hour time-window for thrombolysis. The patient also characterized her deficit as nondisabling, thus extended time-windows for thrombolysis were not pursued. Subsequent magnetic resonance imaging (MRI) of the brain demonstrated multiple small areas of diffusion restriction in bilateral cerebellar hemispheres, including the right superior cerebellum, as well as tiny punctate areas supratentorially in bilateral cerebral hemispheres, compatible with acute ischemic strokes. There was also evidence of chronic infarcts in the left post–central gyrus and right parietal cortex. The patient's telemetry was noted to demonstrate ongoing atrial fibrillation after MRI. Transthoracic echocardiogram did not reveal any significant valvular disease or intracardiac thrombus or mass. Multiple competing mechanisms were suspected as the cause of her ischemic strokes, including cardioembolic (in the setting of atrial fibrillation and NSTEMI) versus multifocal microvascular ischemia due to leukostasis from CML. Dual antiplatelet therapy was stopped, and she was ultimately commenced on therapeutic anticoagulation.

## 3. Discussion

The patient in this vignette developed abrupt onset isolated, high amplitude, low frequency, yes-yes, and postural head tremor without other significant neurological signs or symptoms. Tremor itself can localize to the basal ganglia, cerebellum, or thalamus [7]. The cerebellum plays a role in the cerbellothalamocortical circuits and Guillain–Mollaret triangle (composed of the dentate nucleus, red nucleus, and inferior olivary nucleus) [8]. Functional connectivity involving the dentate nucleus is particularly implicated in tremor severity in Parkinson's disease and essential tremor [9]. Paucity of GABA receptors within the dentate nucleus inversely correlates with progression of essential tremor [10]. Increased synapses between cerebellar climbing fibers and Purkinje cells also lead to greater cerebellar oscillations, which are implicated in the development of kinetic tremor [11].

The differential diagnosis of head tremor is broad and can include entities such as cervical dystonia, essential tremor, Parkinson's disease, and genetic conditions. A head tremor can be seen in cervical dystonia, although these are typically high frequency, irregular tremors, no-no in axis, and persist when patients lie down (unlike the patient presented in this case) [12]. Patients with advanced essential tremor can also develop head tremor, although appendicular tremor precedes head tremor, unlike our case [13]. Head tremor is rarely seen in Parkinson's disease (and our patient did not demonstrate any features of parkinsonism on exam) [14]. While multiple medications are associated with tremor (including neuroleptics, antidepressants, stimulants, steroids, beta-agonists, valproic acid, and tacrolimus, none of which this patient was taking), head tremor is not typically seen in drug-induced tremors. Thyroid dysfunction can result in a subacute onset tremor (however, our patient had normal thyroid stimulating hormone), as can other metabolic disturbances. Genetic tremor syndromes, such as Wilson's disease, can include head tremor but are progressive in onset [7].

Poststroke tremor is generally uncommon, although strokes involving the brainstem and cerebellum can result in a palatal tremor or Holmes tremor [3]. Holmes tremor is typically appendicular; it has been described when there is disruption to the cerebellothalamic, dentatorubroolivary, and nigrostriatal pathways and can be a product of midbrain and pontine infarction (or other lesions) [15, 16]. Palatal tremor (also termed palatal myoclonus) can be seen from lesions to the Guillain–Mollaret triangle resulting in hypertrophic olivary degeneration [17]. Vascular parkinsonism, which can present acutely due to acute ischemia involving the basal ganglia or thalamus, or chronically due to cumulative white matter changes in the striatum and globus pallidus, is another cerebrovascular complication to consider [18]. However, head tremor in the absence of appendicular tremor due to acute stroke is rare. Kim et al.'s [19] case series presents four patients with abrupt onset head tremor (without appendicular tremor). Three of the four patients experienced a stroke involving the paramedian pons and one patient involving the superior cerebellar hemisphere. These patients did demonstrate other focal neurological deficits however [19]. Our case is unique in that the patient's presenting clinical symptom was an isolated head tremor (as opposed to traditional signs/symptoms of posterior circulation stroke syndromes, aside from vague vertiginous sensation). Finsterer et al. [20] described a case of a 67-year-old male experiencing yes-yes head tremor (without appendicular tremor) six weeks after bilateral cerebellar (and right occipital) infarctions [20].

While poststroke tremor can be refractory to medical therapy, clonazepam, propranolol, and levodopa have been trialed for head tremor. Due to our patient's comorbidities and consequent medications (which included metoprolol), these therapies were not trialed for this patient. Holmes tremor also responds to valproic acid. In addition to tremor, the spectrum of poststroke movement disorders includes myoclonus, dystonia, and stereotypies [18].

Our case adds to the growing body of literature implicating the cerebellum in the development of isolated head tremor, and expands upon the spectrum of poststroke movement disorders. Additionally, our patient serves as a reminder to consider acute tremor as a possible manifestation of acute cerebrovascular syndromes.

## Data Availability

Data sharing is not applicable to this article as no datasets were generated or analyzed during the current study.
